# Metataxonomic analysis of endophytic bacteria of blackberry (*Rubus ulmifolius* Schott) across tissues and environmental conditions

**DOI:** 10.1038/s41598-024-64248-5

**Published:** 2024-06-11

**Authors:** Rocío Roca-Couso, José David Flores-Félix, Saptarathi Deb, Lucia Giagnoni, Alessandra Tondello, Piergiorgio Stevanato, Andrea Squartini, Paula García-Fraile, Raúl Rivas

**Affiliations:** 1 Department of Microbiology and Genetics, Biology Departmental Building, University of Salamanca, 37007 Salamanca, Spain; 2 Institute for Agribiotechnology Research (CIALE), 37185 Salamanca, Spain; 3 Department of Agronomy, Animals, Food, Natural Resources, and Environment, DAFNAE University of Padova, 35020 Legnaro, PD Italy; 4 Associated Unit, University of Salamanca-CSIC (IRNASA), 37008 Salamanca, Spain

**Keywords:** Environmental microbiology, Soil microbiology, Microbial communities, Metagenomics

## Abstract

(1) Background: Endophytic bacteria represent an important component of plant wellness. They have been widely studied for their involvement in plant development and enhancement of stress tolerance. In this work, the endophytic communities of roots, stems, and leaves of blackberry (*Rubus ulmifolius* Schott) were studied in three different niches: natural, riverside, and human-impacted niches. (2) Results: The microbiome composition revealed that *Sphingomonadaceae* was the most abundant family in all samples, accounting for 9.4–45.8%. In contrast, other families seem to be linked to a specific tissue or niche. Families *Microbacteriaceae* and *Hymenobacteraceae* increased their presence in stem and leaf samples, while *Burkholderiaceae* abundance was important in riverside samples. Alpha and beta diversity analyses showed that root samples were the most diverse, and they gathered together in the same cluster, apart from the rest of the samples. (3) Conclusions: The analysis of the microbiome of *R. ulmifolius* plants revealed that the composition was essentially the same in different niches; the differences were primarily influenced by plant tissue factors with a core genome dominated by *Sphingomonadaceae*. Additionally, it was observed that *R. ulmifolius* can select its own microbiome, and this remains constant in all tissues evaluated regardless the niche of sampling.

## Introduction

Plants are complex living beings that interact with all the surrounding organisms, including microbial communities from soil, water, and air^1^. In fact, plants possess an internal microbial community that influences their metabolism and development^2^. This community is called the microbiome and is usually referenced as the second genome of the plant^3^. The microbiome is formed by microorganisms that live in the surrounding soil (rhizosphere), on plant surfaces (phyllosphere), and/or inside plant tissues (endosphere)^4^. The last mentioned are endophytes, which are components of the plant microbiota that colonize internal plant tissues and establish nonharmful relationships with their host^5^. This type of microorganisms are usually soil inhabitants which can enter through plant roots. Once inside, they may use the vascular system for travelling and settling in other tissues^6^. However, they can enter the plant by other pathways, such as directly, through wounds or stomata, or even vertically transferred, from seeds^7,8^. Colonization is a variable activity and depends on multiple factors, such as plant species, plant genotype, growth stage, physiological status, type of plant tissue, environmental conditions, and sampling season^9^.

Studies on endophytic bacterial diversity discovered that the vast majority of endophytes (96%) fall under the phyla Actinomycetota, Pseudomonadota, Bacteroidota, and Bacillota^9,10^. Many of the described bacteria are able to synthesize bioactive compounds with antifungal and antibacterial activity, playing an active role in plant defense^11^. Additionally, endophytic bacteria also contribute to other beneficial aspects for their hosts. They are involved in the acceleration of seedling emergence, promotion of plant establishment under adverse conditions, and enhancement of plant growth^12^.

In the last few years, the study of these communities increased, and it was discovered that many of them have biotechnological potential^13^. For example, they can be used as biofertilizers since they increase the productivity and nutritional content of crops^14^; for the synthesis of new drugs of pharmaceutical interest, such as antibiotics or anticancer agents^9,15^; or for phytoremediation, since some of them are resistant to heavy metals and/or are able to produce metabolic compounds that degrade xenobiotics^12,16^.

In that sense, unraveling the microbial communities that inhabit plant tissues may be a useful tool for identifying and establishing a set of culturable bacteria whose industrial potential may be used for future research and application to achieve more sustainable agricultural practices^17^. Endophytic bacteria of different plant tissues have been targeted as a new source of new biofertilizers since it they are involved in improving plant nutrition acquisition and providing protection against biotic and abiotic factors^18^. Currently, only a few plant species were studied relative to their endophytic bacterial community and are well documented, such as rice, wheat, or tomato^12,17–20^; however, crops such as blackberry remain unclear. A necessary technical comment that applies to this work as well as to any other in which plant associated bacteria are targeted by molecular methods, is the following, and will be intended for all instances in which we will use the term endophytes throughout the text. Unlike the case of culture-dependent approaches, a distinction between strictly endophytic bacteria and those colonizing the plant surface (epiphytic) is not achievable by plant surfaces sterilization since DNA from killed bacteria is amplified just as efficiently as that from the internal ones. Washing the specimens before extraction is instead the feasible measure to minimize epiphytic occurrences but since phyllosphere or rhizosphere bacteria can use attachment mechanisms to adhere to the plant epidermis, the term endophytes is broadly also intending a possible share of epiphytes^21^.

In the present study, we focused on blackberries (*Rubus ulmifolius* Schott). These plants recently gained more importance at a social level since their fruits come with benefits for human health, and consequently, industrial production also increased^22^. That is why research on endophyte communities is now needed due to their importance as functional and therapeutic foods. Some studies with related plants were already performed, such as *Rubus fruticosus* L.^7^ or *Vaccinium myrtillus* L.^23^, *Vaccinium vitis-idaea* L., and *Empetrum nigrum* L.^5,22^. However, this research focused on the cultivable portion of endophytes and they do not perform a complete study of endophyte composition. Indeed, a full understanding of blackberry endophytes is still missing. Unraveling the endophytic composition of blackberry may create an advantageous context for the establishment of new biofertilizers for this type of crop and for the discovery of new beneficial microorganisms for the different aspects of human activities.

## Material and methods

### Plant sampling and climate conditions

*R. ulmifolius* individual plants from three different environments in Ciudad Rodrigo, in Salamanca, Spain, were taken for study. A pool of 5 plant individuals was sampled in each location due to the difficulty to differentiate blackberry individual since this bush reproduces by propagation. This area is located under the climatic conditions of type Csa (warm temperate climate with dry and hot summer) according to the Köppen–Geiger classification. Wild plants growing under different conditions were sampled. One niche was considered the natural one (40° 35′ 23.4″ N 6° 29′ 53.6″ W), since it consisted on a distric cambisol under the standard climatic conditions of type Csa (40° 35′ 23.4″ N 6° 29′ 53.6″ W), hereafter referred to as “S”; another niche was on the riverside, so it was influenced by an eutric fluvisol and high humidity (40° 34′ 51.5″ N 6° 30′ 42.2″ W), referred to as “R”; and the last niche was a dystric cambisol area influenced by harvesting activity (40° 35′ 27.7″ N 6° 29′ 51.4″ W), referred to as “H”. For all the niches, a pool of roots, stems, and leaves were sampled and identified with “R”, “S” and “L”, respectively (Table 1).

**Table 1:** Summary of sample nomenclature.

Sample	Tissue	Growth conditions	Coordinates
RS	Root	Natural-conditions	40° 35′ 23.4″ N 6° 29′ 53.6″ W
SS	Stem	Natural-conditions	40° 35′ 23.4″ N 6° 29′ 53.6″ W
LS	Leaf	Natural-conditions	40° 35′ 23.4″ N 6° 29′ 53.6″ W
RR	Root	Riverside-conditions	40° 34′ 51.5″ N 6° 30′ 42.2″ W
SR	Stem	Riverside-conditions	40° 34′ 51.5″ N 6° 30′ 42.2″ W
LR	Leaf	Riverside-conditions	40° 34′ 51.5″ N 6° 30′ 42.2″ W
RH	Root	Human impacted conditions	40° 35′ 27.7″ N 6° 29′ 51.4″ W
SH	Stem	Human impacted conditions	40° 35′ 27.7″ N 6° 29′ 51.4″ W
LH	Leaf	Human impacted conditions	40° 35′ 27.7″ N 6° 29′ 51.4″ W

Sampling was performed during May, being the time when plants start to develop and leaves begin to sprout, indicating the beginning of the reproductive phase. Sampling was performed differently according to the tissue and the used tools were previously sterilized with 70% ethanol. Aerial tissues were cut with scissors, while the root samples were obtained by digging with a gardening shovel in the basal area of the plant and cutting with scissors. The samples were kept in plastic bags and taken to the laboratory at 4 °C.

### DNA extraction and targeted amplicon sequencing

Total DNA extraction was conducted with DNeasy Plant Mini Kit (Qiagen, Germantown, MD, United States) following the manufacturer’s instructions. The concentration of the purified DNA was measured using a Qubit dsDNA HS Assay (Thermo Fisher Scientific, Waltham MA, USA). FastPrep-24 TM 5G (Thermo Fisher Scientific) was used as complementary lysis, using the program 40 s, at 60 m/s, three times. DNA metabarcoding analyses were carried out by using a 16S Ion Metagenomics Kit (Thermo Fisher Scientific), and library preparation was based on two combined pools of primers targeting seven different hypervariable regions (V2–V4–V8 primer pool and V3–V6–V7–V9 primer pool proprietary to Thermo) (Thermo Fisher Scientific). The primer sequences are proprietary information of the manufacturer. Libraries were normalized at equal DNA concentration and 16S rRNA multiamplicon sequencing was performed using an Ion GeneStudio S5 System (Thermo Fisher Scientific).

### Bioinformatic analysis

The analysis was done on the pooled reads as implied by the multi-amplicon approach customary procedure. Primers were removed from raw reads by trimming 20 nucleotides on both ends using the cutadapt utility^24^ in Quantitative Insights Into Microbial Ecology 2 (QIIME2) v2020.08^25^. “Qiime dada2” was used for denoising, chimera removal, and dereplication. The “classify-consensus-blast” plugin using SILVA SSU v138.1^26^ as a reference database was used for taxonomic classification of Amplicon Sequence Variants (ASVs). Then, the Calypso online suite^27^ to Total Sum Scaling (TSS) normalized for library size differences was used to process the taxonomy abundance table at different taxonomic levels. The Centered-Log Ratio (CLR) transformation was adopted to deal with the compositional issues of the datasets in view of the subsequent analyses. Taxa with less than the average of 10 reads across samples were removed from further analysis. Sequences identified as plant chloroplasts and mitochondria were filtered off after annotation.

Abundances, alpha and beta diversities were calculated using the ASV table and by using vegan package from R (www.r-project.org). Community richness (“observed ASV” index, Chao1 index, and ACE index), diversity (Shannon index and Simpson index), and evenness were calculated. The multiple comparison of phyla abundances and alpha diversity indexes among group samples was calculated with Kruskal–Wallis test to compare groups. A post hoc Dunn paired test (5% significance) using a Bonferroni adjustment was performed to determine which levels of the independent variable differ from each other. All statistical analyzes were performed in the Rstudio version 4.1.2.

Beta diversity comparisons of treatments were made following the Bray method^28^ by using the vegan package from Rstudio (www.r-project.org) and permutation ANOVA (pseudo-F, 999 permutations) was used to compare Bray‒Curtis distances). Reads were deposited to GenBank of the NCBI under the SRA accession PRJNA981419.

### Determination of Shared ASVs and Differences in the Abundance of Bacterial Taxa

Specific enrichment or exclusion of microbial taxa were searched over three categories: (1) roots versus stems, (2) roots versus leaves, (3), and stems versus leaves. The analysis of composition of microbiomes (ANCOM) framework^29^. Core microbiome analysis was performed with the “q2_feature_table” plugin and Marker Data Profiling of MicrobiomeAnalyst (https://www.microbiomeanalyst.ca) to predict sequencing data function.

### Plant collection statement

Plant samples were collected under the international and national regulation of Nagoya Protocol with the Internationally Recognized Certificates of Compliance (IRCC) Number: ABSCH-IRCC-ES-266210-1. Plant samples were not stored in private herbarium due to destructive process of the employed methods. Plant species were identified by Dr. José David Flores-Félix.

## Results

### Composition of endobiomes associated with *Rubus ulmifolius*

**Figure 1:**
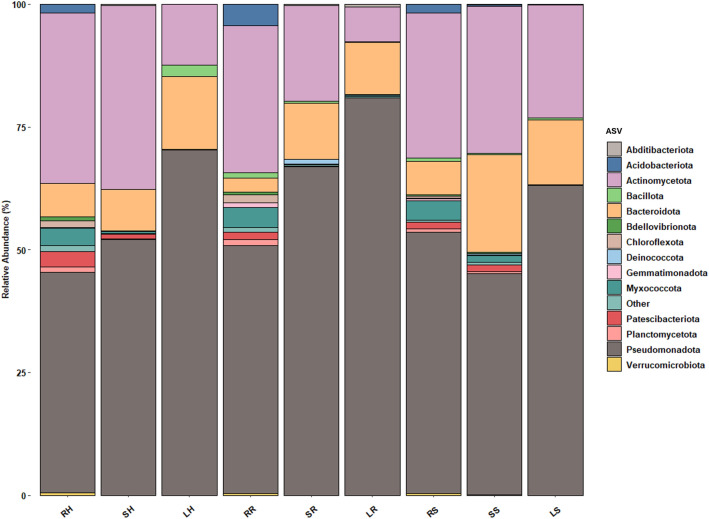
Relative abundance of bacterial phyla in different anatomic parts of the *Rubus ulmifolius* plant sampled in different locations. The figure shows bars of roots, stems, and leaves in human-impacted conditions (RH, SH, LH); of roots, stems and leaves in riverside environments (RR, SR, LR); and of roots, stems and leaves in natural environments (RS, SS, LS).

In this work, endophyte communities were characterized by sequencing 16S amplicons obtained from DNA extracted from roots, stems, and leaves from wild individuals of *R. ulmifolius*. 16S amplicon processing consisted of removing low-quality reads, in terms of base-calling accuracy score and length, chimeras, and samples with a low depth of coverage, resulting in a dataset of 1,019,239 high-quality reads (median reads per sample = 37,750) (Table 2). Sample SH2 was removed from forward analyses since it produced zero reads. After filtration, the number of ASVs among the samples was 29–1968, which mainly belonged to three phyla, Pseudomonadota, Actinomycetota, and Bacteroidota, which accounted for 53.7, 29.1, and 8.3% of all sequencing reads collectively considered, respectively (Fig. 1).

**Table 2:** Metadata, number of reads and ASVs of bacterial communities present in the samples.

Sample	Tissue	Growth conditions	Raw reads	% plant organelles	Reads after processing*	Observed ASVs
RS1	Root	Natural-conditions	81,962	55.5	30,632	1039
RS2	Root	Natural-conditions	136,198	23.7	75,855	1968
RS3	Root	Natural-conditions	226,675	34.0	106,290	1794
SS1	Stem	Natural-conditions	76,997	73.8	18,922	390
SS2	Stem	Natural-conditions	116,674	0.7	84,176	1514
SS3	Stem	Natural-conditions	101,690	65.7	30,755	416
LS1	Leaf	Natural-conditions	106,636	53.7	38,283	414
LS2	Leaf	Natural-conditions	76,952	84.7	11,542	139
LS3	Leaf	Natural-conditions	66,264	80.9	12,236	203
RR1	Root	Riverside-conditions	124,070	54.0	48,817	1530
RR2	Root	Riverside-conditions	165,562	24.2	89,329	1719
RR3	Root	Riverside-conditions	89,235	62.3	28,429	977
SR1	Stem	Riverside-conditions	468,852	94.3	29,000	439
SR2	Stem	Riverside-conditions	115,724	67.2	31,767	523
SR3	Stem	Riverside-conditions	92,629	78.3	18,555	293
LR1	Leaf	Riverside-conditions	107,478	71.2	26,070	311
LR2	Leaf	Riverside-conditions	73,909	76.9	15,737	259
LR3	Leaf	Riverside-conditions	90,918	75.7	19,425	260
RH1	Root	Human-impacted conditions	114,822	25.5	63,677	1407
RH2	Root	Human-impacted conditions	139,924	27.1	75,522	1657
RH3	Root	Human-impacted conditions	108,255	33.0	58,272	1062
SH1	Stem	Human-impacted conditions	111,550	31.0	60,575	825
SH2	Stem	Human-impacted conditions	12	100.0	0	0
SH3	Stem	Human-impacted conditions	85,077	99.2	35,595	470
LH1	Leaf	Human-impacted conditions	117,194	94.9	6085	142
LH2	Leaf	Human-impacted conditions	123,238	99.2	1061	55
LH3	Leaf	Human-impacted conditions	74,267	96.6	2632	81

*After filtering (< 800 nt > 1300 nt), chimera, mitochondria and chloroplast removal.

All the samples had a high content of Pseudomonadota, Actinomycetota, and Bacteroidota, which accounted for 91.1% of the total reads. Specifically, Pseudomonadota represented 44.9, 52.1, and 70.3% in roots, stems, and leaves of human-impacted samples; 50.5, 66.9, and 80.9% in riverside samples; and 53.1, 45.1, and 63.1% in natural samples. Actinomycetota represented 34.6, 37.4, and 12.4% in roots, stems, and leaves of human-impacted samples; 29.9, 19.5, and 7.1% in riverside samples; and 29.5, 30.0, and 23.0% in natural samples. Finally, Bacteroidota represented 6.7, 8.5, and 14.9% in roots, stems, and leaves of human-impacted samples; 2.9, 11.4, and 10.6% in riverside samples; and 6.8, 11.4, and 13.2% in natural samples. Additionally, root samples possessed a great representation of other phyla, such as Myxococcota, which represents 4.1, 4.0, and 3.5% of riverside, natural, and human impacted samples, or Acidobacteriota, which represents 4.4, 1.7, and 1.8%, respectively (Fig. 1).

**Figure 2:**
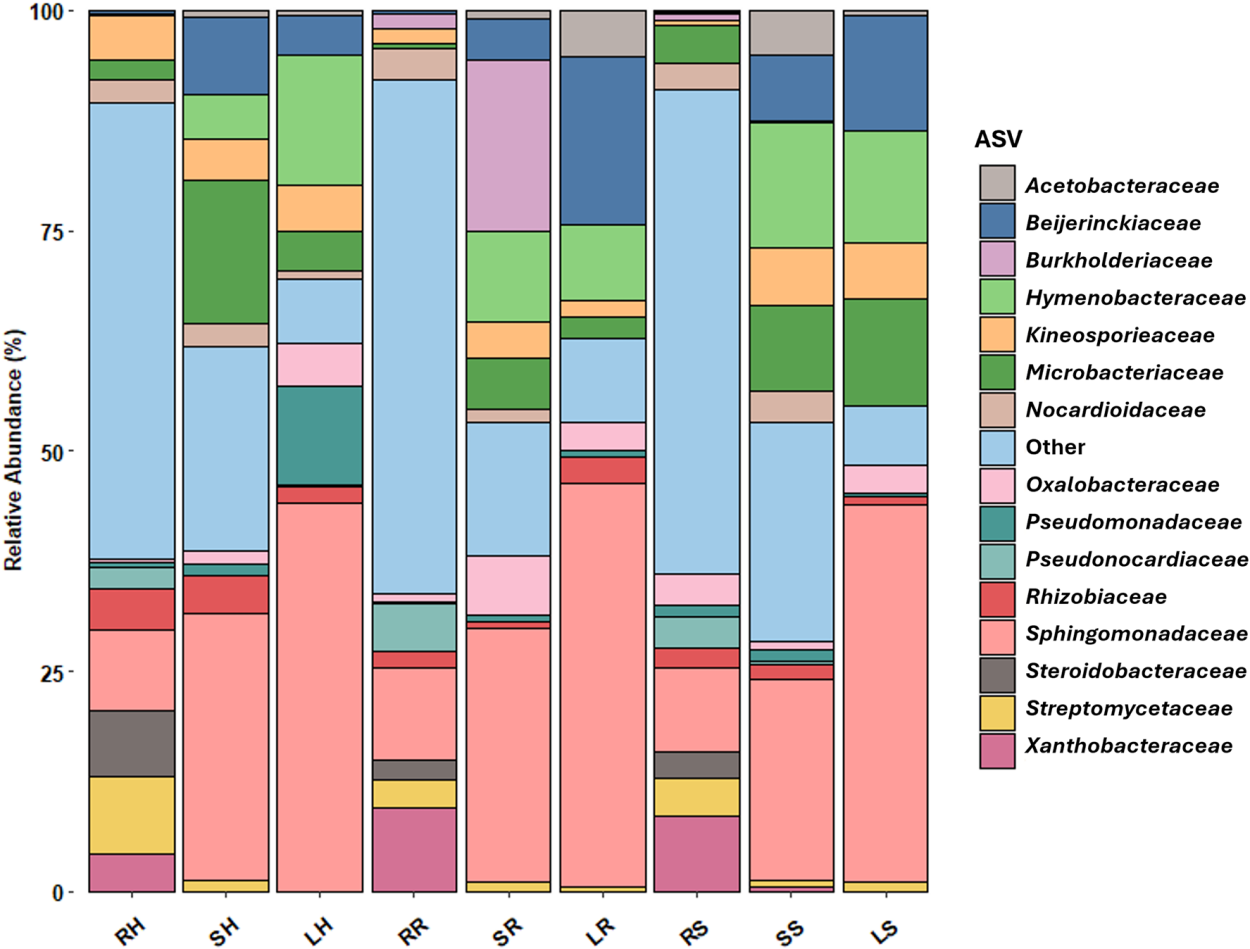
Relative abundance of bacterial families in different anatomic parts of the *Rubus ulmifolius* plant sampled in different locations. The figure shows bars of roots, stems, and leaves in human-impacted conditions (RH, SH, LH); of roots, stems and leaves in riverside environments (RR, SR, LR); and of roots, stems and leaves in natural environments (RS, SS, LS).

Regarding families (Fig. 2), *Sphingomonadaceae* was the most abundant, representing 9.4, 30.4, and 44.1% in roots, stems, and leaves of human-impacted samples; 10.4, 28.8, and 45.8% in riverside samples; and 9.5, 22.8, and 42.8% in natural samples. However, the following most abundant families differed among samples. In natural samples, *Xanthobacteraceae* and *Microbacteriaceae* were the next most abundant, with abundances of 8.6 and 4.3%, respectively. Stem samples showed a decrease in *Xanthobacteraceae* to 0.5%, while *Microbacteriaceae* and *Hymenobacteraceae* increased to 9.6 and 14.4%, respectively. In leaves, *Microbacteriaceae* still increased to 12.0%, while *Hymenobacteraceae* maintained at 12.8%. In riverside samples, *Xanthobacteraceae* and *Pseudonocardiaceae* were the second and third most abundant in roots, with 9.5 and 5.4%, respectively. In stems, there was a decrease in both families to 0.1 and 0.0%, respectively. However, there was again an increase in *Microbacteriaceae*, *Burkholderiaceae,* and *Hymenobacteraceae* to 5.8, 19.4, and 10.3%, respectively. However, in leaves, the only family that maintained was *Hymenobacteraceae* at 8.5%, and *Beijerinckiaceae* and *Acetobacteraceae* increased to 19.0 and 5.4%, respectively. Finally, in human-impacted samples, in roots, the main families were *Streptomycetaceae* and *Steroidobacteraceae,* with abundances of 8.7 and 7.5%, respectively. However, those families decreased in stems until 1.2 and 0.0%, while *Microbacteriaceae* and *Beijerinckiaceae* increased until 16.3 and 8.9%, respectively. In leaves, these two families slightly remained at 4.5 and 4.5%, whereas *Hymenobacteraceae* and *Pseudomonadaceae* increased until 14.8 and 11.1%, respectively. Finally, fraction “Others” gave some interesting results too. Root and stem samples had a huge percentage of “Other” families while in leaves samples was smaller. In root samples of all niches, the fraction “Other” was formed by 200–231 different families, while stems comprised 59–138 families and leaves 22–29 families. However, in all samples most of these families represented less than 1% or even less than 0.1% of the total amount of reads. Thus, root, stem, and leaves of riverside samples, families with less than 1% represented the 89.4,85.1, and 74.4% respectively; in root, stem, and leaves of riverside samples of natural samples, they represented the 85.4, 86.3, and 75.7% respectively; and in root, stem, and leaves of human-impacted samples, 87.9, 78.4, and 79.5% respectively. However, there was a few families that were not plotted in the Fig. 2 whose presence was slightly important, such as *Haliangiaceae* and *Caulobacteraceae* which appeared in riverside and human-impacted roots in a 2%, approximately; *Acetobacteraceae*, which appeared in riverside leaves, and natural stems, around 5%; *Comamonadaceae*, which appeared in all samples between 0.9 and 4.4%; or *Thermomonosporaceae* in human-impacted roots at 3.4%.

**Figure 3:**
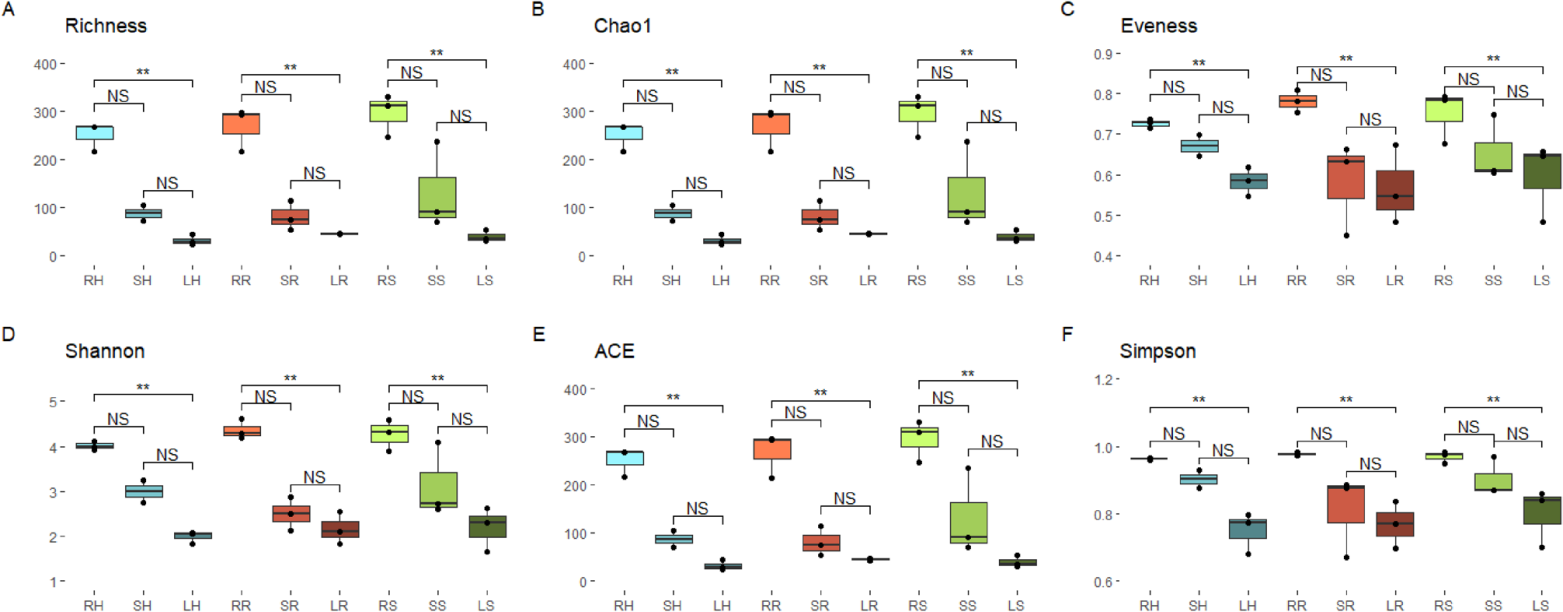
Comparison of alpha diversity; (**A**) ASV richness/observed ASVs; (**B**) boxplots representing the Chao-1 index; (**C**) ACE index; (**D**) Evenness index; (**E**) Shannon index; (**F**) Simpson index for the different samples. Dunn’s pairwise Z; NS: Not significant.

There were statistically significant differences in the abundance of endophytes among samples. The most difS1ferent were leaf and root samples from the riverside environment, where there were differences in eight phyla and seven families. Similarly, leaf, and root samples from the natural environment showed differences in five phyla and five families. Human-impacted root and leaf samples showed differences in four phyla and four families (Table S1). Six alpha diversity indexes were calculated per sample (Fig. 3). In all of them, root samples had the highest values, followed by stem samples and leaf samples. Pairwise analyses found statistically significant differences (Table S2).

**Figure 4:**
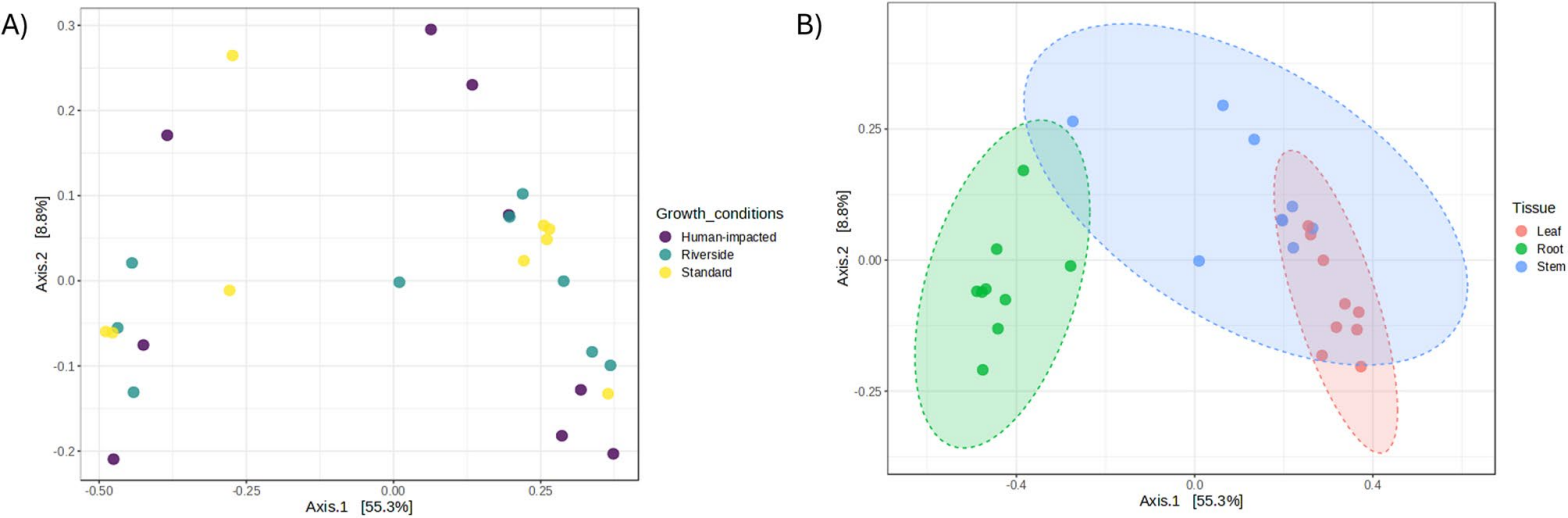
PCoA plot analyzing the relationship between microbiome samples and the distribution of different bacterial families. (**A**) Bacterial communities are clustered by location rather than by (**B**) tissue.

Additional analyses were conducted to investigate potential relationships between different samples types. To achieve this, PCoA plots were created using two components to display differences in bacterial community composition changes among samples (Fig. 4). Statistical differences among plant tissues and locations were measured under PERMANOVA analysis (*p* values ≤ 0.01). Samples are easily clustered by tissue, but there is no ordination when it is analyzed by location (Table 3). Adonis2 analyses were performed to measure the contribution of each variable to the microbial community, supporting the theory that communities are shaped by tissue (R^2^ = 0.56; Pr(> F) = 0.001) rather than location (R^2^ = 0.06; Pr(> F) = 0.676).

**Table 3:** Results of pairwise PERMANOVA analysis.

Pair	F-value	R-squared	*P*-value	FDR
Leaf versus root	31.9067	0.666017	0.001	0.001
Leaf versus stem	4.61482	0.223859	0.001	0.001
Root versus stem	12.5722	0.440015	0.001	0.001

The multi-testing adjustment is based on Benjamini–Hochberg procedure (FDR).

### Relative and absolute abundances of some bacterial taxa associate with the anatomical part of the plant and the geographical location

**Figure 5:**
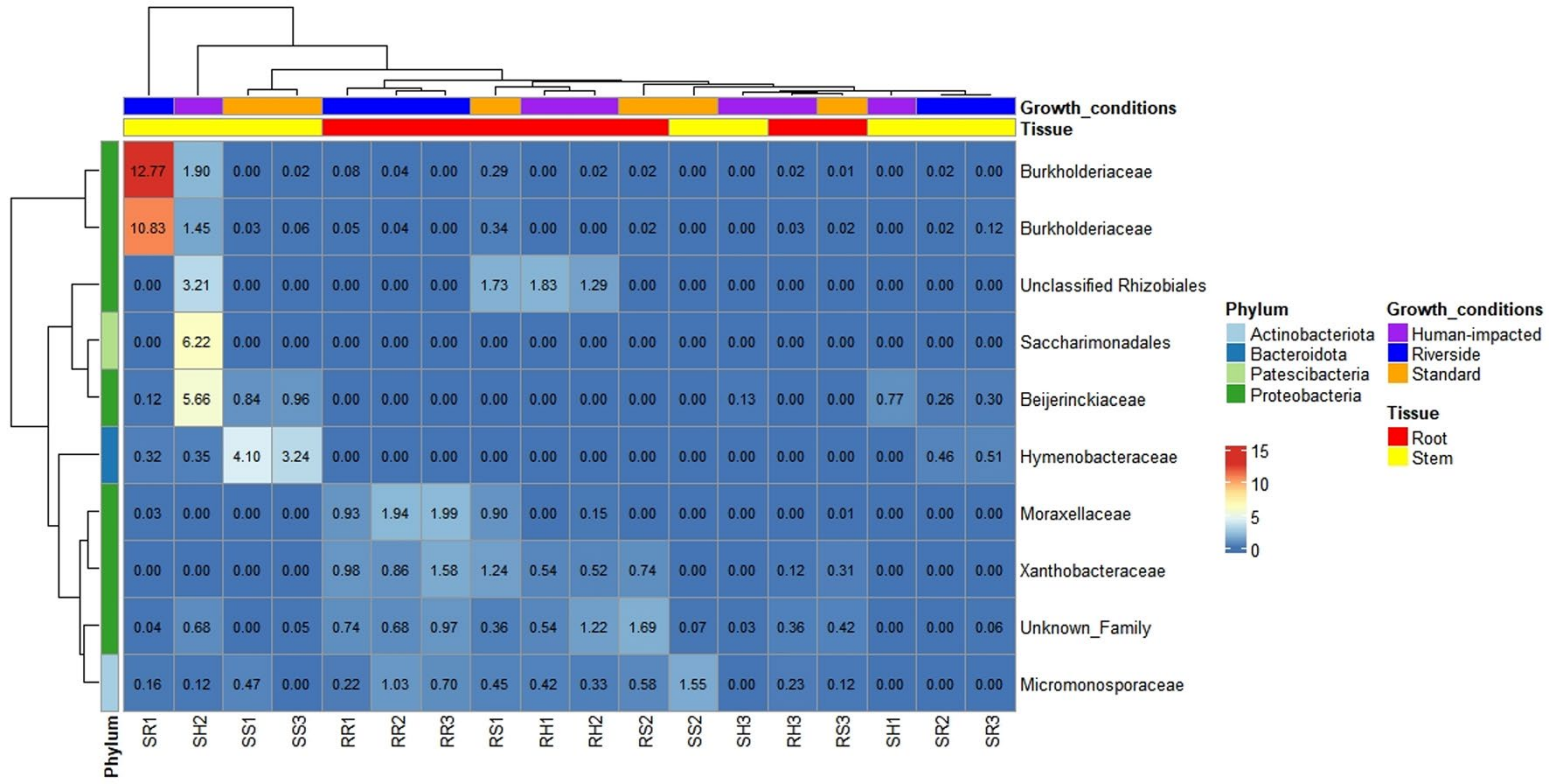
Heatmap showing ANCOM results of differentially abundant family for roots and stems in three growth conditions (Natural, Riverside and Human impacted). Taxa were selected using a family relative abundance at least 0.9% for all samples. Colored squares indicate higher relative abundance for bacterial families in each sample. Similarity between samples and taxa is indicated by dendrograms on each axis.

**Figure 6:**
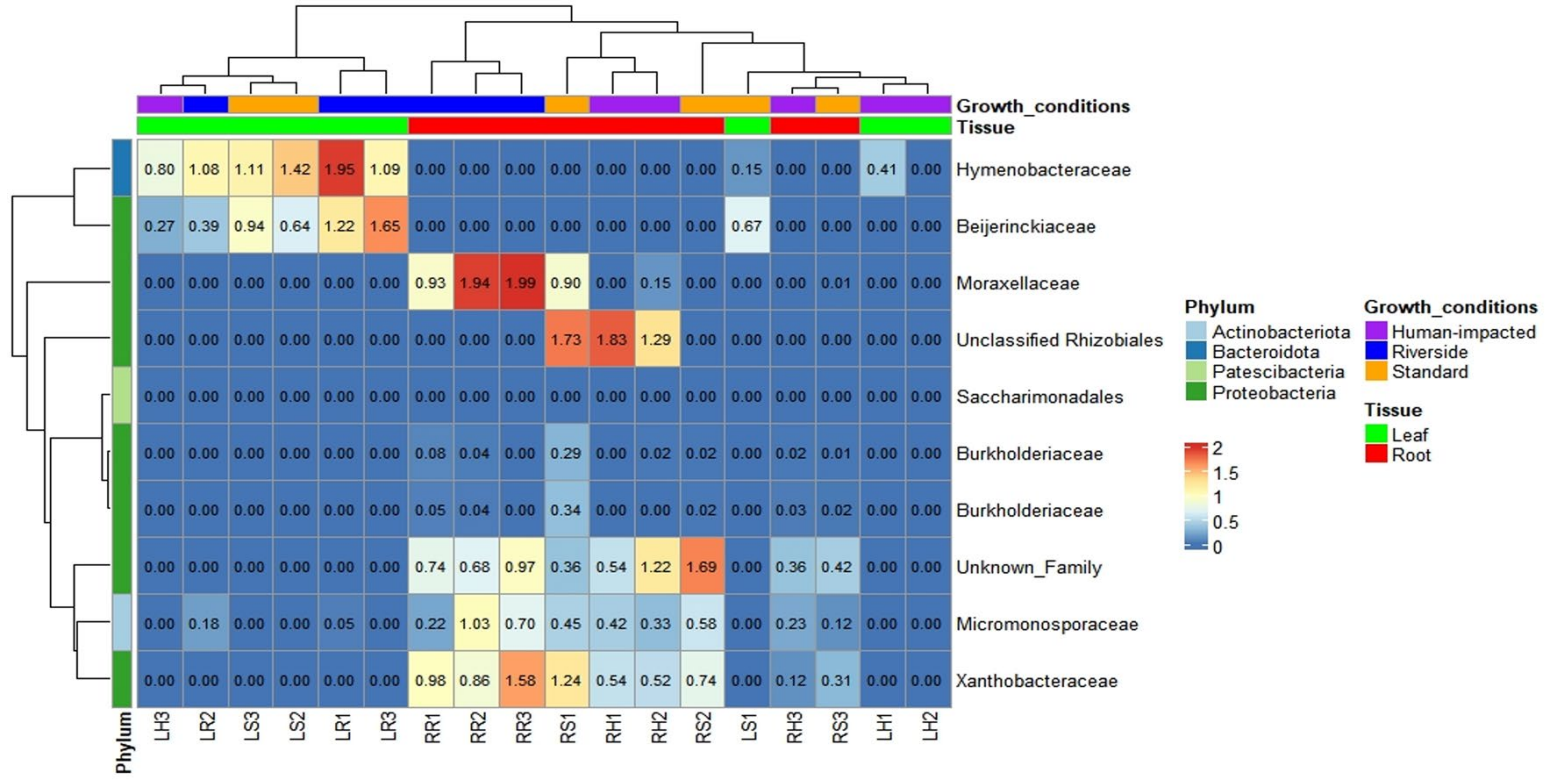
Heatmap showing ANCOM results of differentially abundant family for roots and leaves in three growth conditions (Natural, Riverside and Human impacted). Taxa were selected using a family relative abundance at least 0.9% for all samples. Colored squares indicate higher relative abundance for bacterial families in each sample. Similarity between samples and taxa is indicated by dendrograms on each axis.

**Figure 7:**
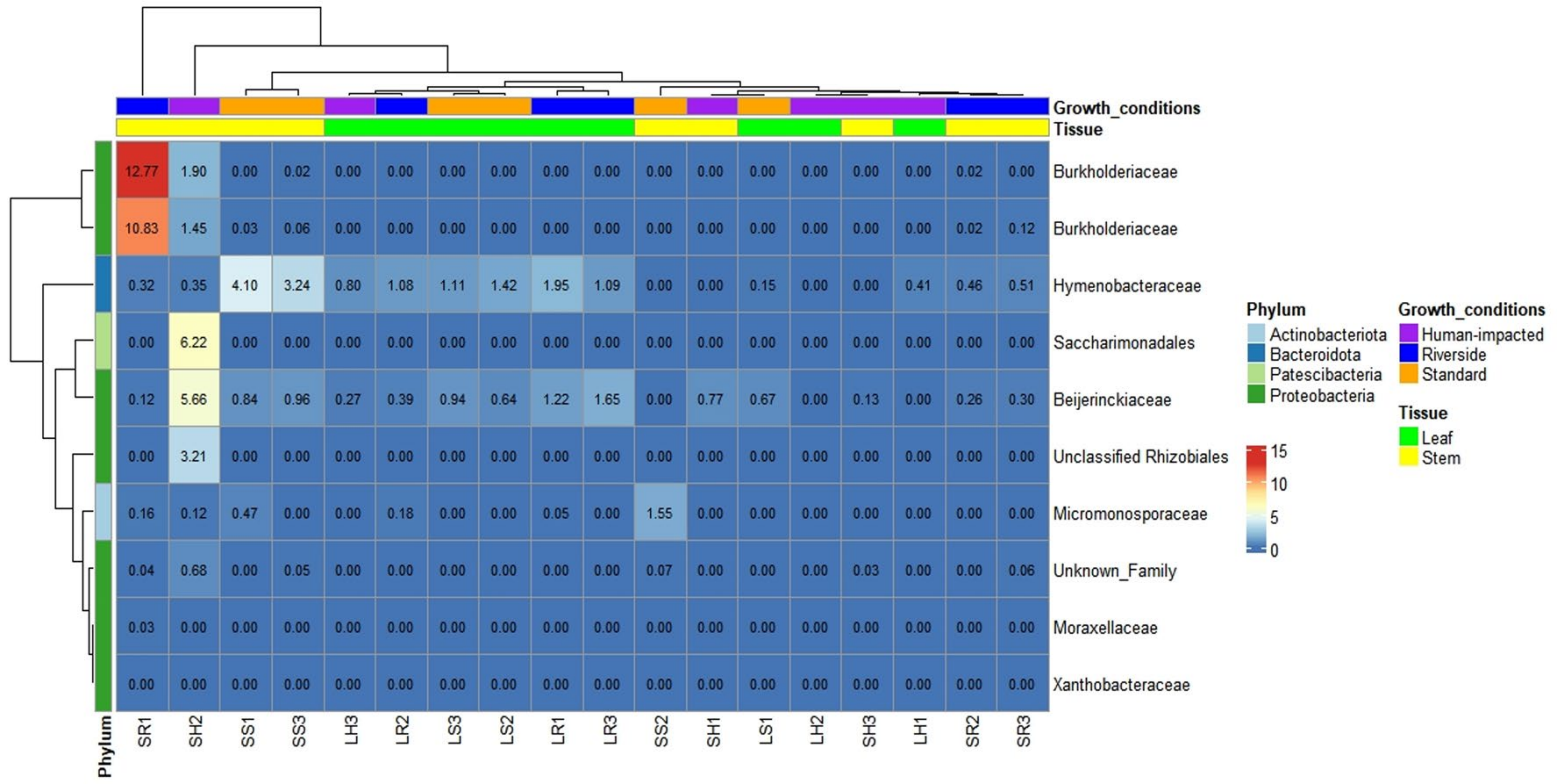
Heatmap showing ANCOM results of differentially abundant family for leaves and stems in three growth conditions (Natural, Riverside and Human impacted). Taxa were selected using a family relative abundance at least 0.9% for all samples. Colored squares indicate higher relative abundance for bacterial families in each sample. Similarity between samples and taxa is indicated by dendrograms on each axis.

Significant enrichments of microbial taxa in different tissues were studied. The results showed no significant differences among locations but tissues. Additionally, differences were clearest when comparing roots versus leaves, while comparing roots versus stems and stems versus leaves showed slightly differences. The ANCOM software was used to identify the bacterial taxa responsible for these differences. Regarding roots and stems, although there are no significant differences, it is observed a slightly increase of *Xanthobacteraceae* bacteria in all root samples. Similarly when comparing stems and leaves samples, no taxa appeared to be of differential importance. However, when comparing roots and leaves, several taxa were found to have differential abundances, as demonstrated by alpha and beta diversities. Leaves samples showed a high abundance of *Hymenobacteraceae* in all locations and *Beijerinckiaceae* in leaves samples from the riverside location and natural location. While in roots, there is high abundance of *Micromonosporaceae*, *Xanthobacteraceae,* and an unknown family belonging to Pseudomonadota. Specifically there was a considerable increment of *Moraxellaceae* in root samples from the riverside location (Figs. 5, 6, and 7).

**Figure 8:**
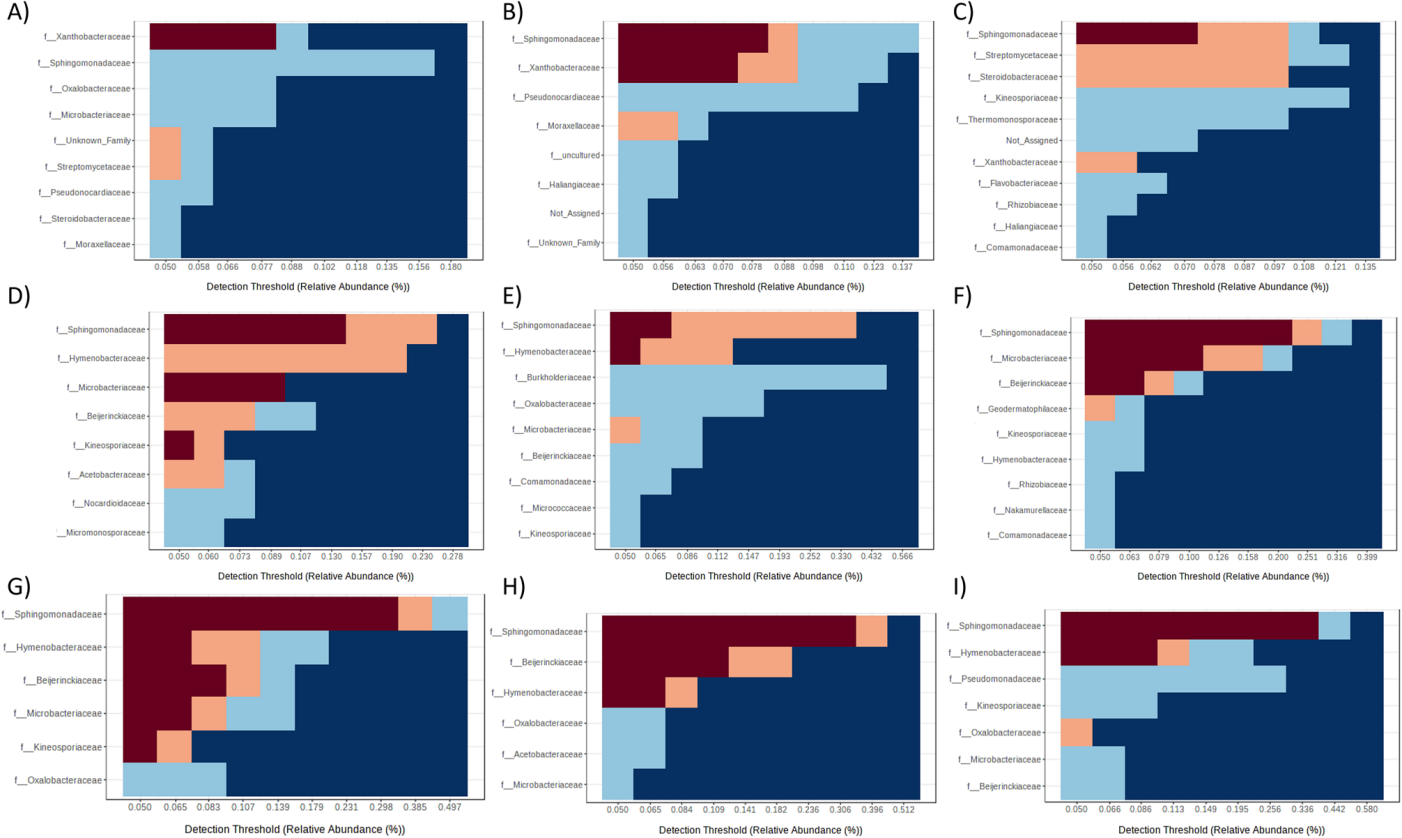
Heatmap of the core microbiome analysis to identify core taxa at family level. Only ASVs with relative abundance > 0.05% and sample prevalence > 20% are shown. The y-axis represents the prevalence level of core features across the detection threshold (relative abundance) range on x-axis. The variation of prevalence of each family is indicated by a gradient of color from blue (decreased) to red (increased). (**a**) RS samples; (**b**) RR samples; (**c**) RH samples; (**d**) SS samples; (**e**) SR samples; (**f**) SH samples; (**g**) LS samples; (**h**) LR samples; (**i**) LH samples.

A more exhaustive investigation was conducted to analyze which genera were specific for each environment. Changes in the absolute abundance of the common bacterial taxa at the family level were revealed by computing the core microbiome of root, stem, leaves, human-impacted, riverside, and natural samples independently. The heat-map representation of the results showed that the most abundant family in all groups was *Sphingomonadaceae* (Fig. 8). The results of DA analysis, *Xanthobacteriaceae* is only prevalent in root samples. In the same way, *Moraxellaceae*, which seemed to be abundant in DA analysis, belongs to core microbiome of RR samples. Three families, *Hymenobacteraceae*, *Microbacteraceae,* and *Beijerinckiaceae*, showed to belong to the core microbiome of all stem and leaf samples. Finally, *Oxallobacteriaceae* only appears in leaves core microbiome. In general, stems, and leaves samples share more taxa than any other two groups.

## Discussion

Recently, a previous work on *R. ulmifolius* studied the endophytic bacterial communities of rhizospheric and root samples^30^. In our work, we extended the study to stems and leaves. At the phylum level, the microbiome composition results of both studies were similar. Pseudomonadota was the most abundant phylum in all samples, 37.0–76.8% in the root samples from the first study and 50.0% in this research. Regarding families, in the previous work, *Xanthomonadaceae* was the most abundant family in roots, followed by *Solibacteraceae*, *Chitinophagaceae,* and *Micropepsaceae*. Here, *Xanthomonadaceae* represented only 7.5% of the total root microbiome, and the rest of the mentioned families represented less than 1%. However, *Xanthomonadaceae* seemed to be one of the most important bacteria in roots, driving differences among samples. A recent study of bacterial communities in root pseudonodules of wild *R. ulmifolius* found 90% of the endophytes were Pseudomonadota, concretely *Stenotrophomonas* (55%)^31^. Although they cannot compare, in our case Pseudomonadota was indeed the main phylum in root samples, accounting for 56.2, 66.1, and 53.8% in human-impacted, riverside, and natural samples respectively, while *Stenotrophomonas* was not identified at all.

It is well known that plants can influence their own microbiomes, recruiting specific groups to colonize their tissues^32^. Soils are one of the most heterogeneous environments, and their diversity usually exceeds that of other habitats^33^. However, although soils possess high diversity, it was reported that all this diversity is concentrated in just a few phyla (Acidobacteriota, Actinomycetota, Bacteroidota, Chloroflexota, Bacillota, and Pseudomonadota) in several studied rhizospheres^34^. Therefore, plants are capable of selectively recruiting specific bacterial groups to interact with them. Similarly, research on root diversity across different plant species showed an enrichment of specific phyla, mainly Actinomycetota, Bacteroidota, and Pseudomonadota, while others, such as Acidobacteriota, decrease^35^. Although, this phylum is not tipically identified as endophytic, it still represents 0.7% of identified endophytes^36^. However, it was observed that they are unable to colonize, likely due to shifts in pH, O_2_ and nutrient content^37^.

In this study, a similar effect appears. Alpha diversity analyses (Fig. 3) revealed that root samples were the most diverse, since diversity decreased from root to leaf samples. Moreover, stem and leaf samples showed an enrichment of some families that are less represented in root samples. Specifically, the Pseudomonadota concentration increased from 49.5% in roots to 55.0% and 71.5% in stems and leaves, respectively. This effect was studied in other crops, showing that Pseudomonadota is in general the most abundant phylum in endophytic plant microbiomes since it is usually selected by plants^36^. Gammaproteobacteria are those that increase the most. This was also reflected in previous studies, where they represented 47% of potato and 30 to 98% of rice endospheres^37^. In the case of the studied plants, this class is quite abundant in roots, representing 15.8% in human-impacted samples, 20.8% in riverside samples and 22.7% in natural samples. However, in both human-impacted and natural stem samples, the content decreases to 7.4 and 6.2%, while in riverside stem samples increases to 31.1%. The reasons why this group of bacteria may be selected remain unknown; however, their presence was positively related to plant immunostimulatory activity^38^. Alphaproteobacteria represent a great percentage of the root bacterial community composition. The same occurred in *R. ulmifolius* root samples, and in this case, this trend remained consistent across all the three environments, with an increase in stems and leaves. Alphaproteobacteria were also studied as one of the most represented groups inside plants. It was revealed that plant-associated and plant symbiotic members share a core of genes that includes a set related to the carbohydrate transport and metabolism category needed to establish nitrogen-fixing symbiosis^39^.

Other phyla that usually increase in other plant roots are Actinomycetota and Bacteroidota^40^. Actinomycetota are known to possess several activities that may benefit plant growth. Those are the plant growth promoting (PGP) activities, and several members of this phylum were studied for this role. For example, over 20% of Actinomycetota are able to solubilize phosphate forms, allowing plants to obtain that nutrient. They are also related to nitrogen fixing activities^41^. In the same way, Bacteroidota are usually associated with PGP activities and protection against pathogens^42^.

These results support the theory by which plants can select which bacteria can colonize their tissues^43^. Selection mechanisms remain unknown; however, some approaches were described that involve plant‒bacteria and bacteria‒bacteria interactions and the contribution of the immune system of plants^38,43^. Most of plant endophytic bacteria came from the surrounding soil and some are able to travel through the vascular system and settle in other organs^44^. Thus, endophytic bacteria may establish a symbiotic relationship with plants and provides them with nutrients, water, protection from herbivory and systemic resistance against various biotic and abiotic stress factors^45^. Then, it seems plausible that in blackberry, bacteria are able to colonize plants through roots and travel to reach stem and leaves. This is visible since diversity is higher in the root samples and decrease the highest the organ is. Moreover, when diversity is compared between organs, there are no statistically differences between adjacent organs, such as root and stems, and stems and leaves. Significant differences are only found when roots and leaves are compared. This may indicate that colonization from roots to leaves is staggered and each organ represent a barrier for the bacteria to reach the upper^46^.

Furthermore, the selection of the endophytic populations prevails over environmental conditions and those imposed by human activities (Fig. 3, Table 3). The obtained results support the idea that plants have a significant impact on the recruitment and selection of the soil microbiome^47^. These data suggest that major perturbations, such as climate change, contaminant introduction or changes in biogeochemical cycles, are required to induce plant microbiome changes^48^ not just land use changes within a similar climatic/environmental system. Moreover, comprehending the core and accessory microbiomes of wild species related to domestic species like *R. ulmifolius* can aid in the development of innovative biofertilization strategies to enhance crop adaptation, resilience, and the functionality of their microbiomes^18^. This phenomenon is attributed to the fact that domestication processes are linked to a reduction in the diversity, complexity, and functionality of rhizospheric and endophytic microbiomes, which can lead to a decrease in certain traits such as pathogen resistance and stress resilience^49^. Therefore, the selection of core components from the microbiome of wild species may represents an effective strategy to enhance plant fitness through their microbiome in the future.

## Conclusions

In conclusion, 16S rRNA profiling of the *Rubus ulmifolius* plant microbiome revealed that its composition was essentially the same across different environments and that the differences in composition were primarily influenced by plant tissue factors. However, despite the different microbiome composition across tissues in the same plant, a core of taxa is maintained, where *Sphingomonadaceae* is clearly dominant. Additionally, it was observed that *R. ulmifolius* is able to select its own microbiome, and this remains constant in all the evaluated tissues regardless of conditions . This is the first attempt to study the composition of endophytic bacteria inside different tissues of *R. ulmifolius* plants growing in different environments. This study contributes to building a foundation for future studies with the aim of blackberry plants as a source of potential biocompound alternatives.

### Supplementary Information

Supplementary Information.

## Data Availability

Reads were deposited to GenBank of the NCBI under the SRA accession PRJNA981419.

## References

[1] Knelman JE, Graham EB, Prevéy JS, Robeson MS, Kelly P, Hood E, Schmidt SK (2018). Interspecific plant interactions reflected in soil bacterial community structure and nitrogen cycling in primary succession. Front. Microbiol..

[2] Yang R, Liu P, Ye W (2017). Illumina-based analysis of endophytic bacterial diversity of tree peony (Paeonia Sect. Moutan) roots and leaves. Braz. J. Microbiol..

[3] Riva V, Mapelli F, Bagnasco A, Mengoni A, Borin S (2022). A meta-analysis approach to defining the culturable core of plant endophytic bacterial communities. Appl. Environ. Microbiol..

[4] Trivedi P, Leach JE, Tringe SG, Sa T, Singh BK (2020). Plant–microbiome interactions: From community assembly to plant health. Nat. Rev. Microbiol..

[5] Nguyen MP, Lehosmaa K, Martz F, Koskimäki JJ, Pirttilä AM, Häggman H (2021). Host species shape the community structure of culturable endophytes in fruits of wild berry species (*Vaccinium myrtillus* L., *Empetrum nigrum* L. and *Vaccinium vitisidaea* L.). FEMS Microbiol. Ecol..

[6] Bulgari D, Casati P, Quaglino F, Bianco PA (2014). Endophytic bacterial community of grapevine leaves influenced by sampling date and phytoplasma infection process. BMC Microbiol..

[7] Contreras M, Loeza PD, Villegas J, Farias R, Santoyo G (2016). A glimpse of the endophytic bacterial diversity in roots of blackberry plants (*Rubus fruticosus*). Genet. Mol. Res..

[8] White JF (2019). Review: Endophytic microbes and their potential applications in crop management. Pest Manag. Sci..

[9] Gouda S, Das G, Sen SK, Shin HS, Patra JK (2016). Endophytes: A treasure house of bioactive compounds of medicinal importance. Front. Microbiol..

[10] Khan SS, Verma V, Rasool S (2020). Diversity and the role of endophytic bacteria: A review. Botanica Serbica.

[11] Roca-Couso R, Flores-Félix JD, Rivas R (2021). Mechanisms of action of microbial biocontrol agents against *Botrytis cinerea*. J. Fungi.

[12] Ryan RP, Germaine K, Franks A, Ryan DJ, Dowling DN (2007). Bacterial endophytes: Recent developments and applications. FEMS Microbiol. Lett..

[13] Burragoni SG, Jeon J (2021). Applications of endophytic microbes in agriculture, biotechnology, medicine, and beyond. Microbiol. Res..

[14] Ayuso-Calles M, García-Estévez I, Jiménez-Gómez A, Flores-Félix JD, Escribano-Bailón MT, Rivas R (2020). *Rhizobium laguerreae* improves productivity and phenolic compound content of lettuce (*Lactuca sativa* L.) under saline stress conditions. Foods.

[15] Guo B, Wang Y, Sun X, Tang K (2008). Bioactive natural products from endophytes: A review. Appl. Biochem. Microbiol..

[16] Syranidou E, Christofilopoulos S, Gkavrou G, Thijs S, Weyens N, Vangronsveld J, Kalogerakis N (2016). Exploitation of endophytic bacteria to enhance the phytoremediation potential of the wetland helophyte *Juncus acutus*. Front. Microbiol..

[17] Agnolucci M, Palla M, Cristani C, Cavallo N, Giovannetti M, De AM, Gobbetti M, Minervini F (2019). Beneficial plant microorganisms affect the endophytic bacterial communities of durum wheat roots as detected by different molecular approaches. Front. Microbiol..

[18] Afridi MS (2022). New opportunities in plant microbiome engineering for increasing agricultural sustainability under stressful conditions. Front. Plant Sci..

[19] Zhao J, Zhao X, Wang J, Gong Q, Zhang X, Zhang G (2020). Isolation, identification and characterization of endophytic Bacterium *Rhizobium oryzihabitans* sp. Nov., from rice root with biotechnological potential in agriculture. Microorganism.

[20] Cochard B, Giroud B, Crovadore J, Chablais R, Arminjon L, Lefort F (2022). Endophytic PGPR from tomato roots: Isolation, in vitro characterization and in vivo evaluation of treated tomatoes (*Solanum lycopersicum* L.). Microorganisms.

[21] Saito A, Ikeda S, Ezura H, Minamisawa K (2007). Microbial community analysis of the phytosphere using culture-independent methodologies. Microbes Environ..

[22] Brennan RM (2014). Berry crops. Hortic. Plants People Places.

[23] Gonçalves AC, Sánchez-Juanes F, Meirinho S, Silva LR, Alves G, Flores-Félix JD (2022). Insight into the taxonomic and functional diversity of bacterial communities inhabiting blueberries in Portugal. Microorganisms.

[24] Martin M (2011). Cutadapt removes adapter sequences from high-throughput sequencing reads. EMBnet. J..

[25] Bolyen E (2019). Reproducible, interactive, scalable and extensible microbiome data science using QIIME 2. Nat. Biotechnol..

[26] Quast C, Pruesse E, Yilmaz P, Gerken J, Schweer T, Yarza P, Peplies J, Glöckner FO (2013). The SILVA ribosomal RNA gene database project: Improved data processing and web-based tools. Nucleic Acids Res..

[27] Zakrzewski M, Proietti C, Ellis JJ, Hasan S, Brion MJ, Berger B, Krause L (2017). Calypso: A user-friendly web-server for mining and visualizing microbiome-environment interactions. Bioinformatics.

[28] Roger BJ, Curts JT (1957). An ordination of the upland forest communities of southern Wisconsin. Ecol. Monogr..

[29] Lin H, Das PS (2020). Analysis of compositions of microbiomes with bias correction. Nat. Commun..

[30] Saati-santamaría Z, Vicentefranqueira R, Kolařik M, Rivas R. In press. Microbiome specificity and fluxes between two distant plant taxa in Iberian forests. , 1–16. 10.1186/s40793-023-00520-x PMC10363313 37481564

[31] Farda B, Mattedi A, Djebaili R, Pace L, Del Gallo M, Pellegrini M (2022). Microbial community investigation of wild brambles with root nodulation from a calcareous nitrogen-deficient soil. Soil Syst..

[32] Jones P, Garcia BJ, Furches A, Tuskan GA, Jacobson D (2019). Plant host-associated mechanisms for microbial selection. Front. Plant Sci..

[33] Daniel R (2005). The metagenomics of soil. Nat. Rev. Microbiol..

[34] Bulgarelli D, Schlaeppi K, Spaepen S, Van Themaat EVL, Schulze-Lefert P (2013). Structure and functions of the bacterial microbiota of plants. Annu. Rev. Plant Biol..

[35] Gottel NR (2011). Distinct microbial communities within the endosphere and rhizosphere of populus deltoides roots across contrasting soil types. Appl. Environ. Microbiol..

[36] Hardoim PR (2015). The hidden world within plants: Ecological and evolutionary considerations for defining functioning of microbial endophytes. Microbiol. Mol. Biol. Rev..

[37] Liu H, Carvalhais LC, Crawford M, Singh E, Dennis PG, Pieterse CMJ, Schenk PM (2017). Inner plant values: Diversity, colonization and benefits from endophytic bacteria. Front. Microbiol..

[38] Kalpana K, Montenegro D, Romero G, Peralta X, Oksuz BA, Heguy A, Tsuji M, Kawamura A (2019). Abundance of plant-associated gammaproteobacteria correlates with immunostimulatory activity of angelica sinensis. Medicines.

[39] Pini F, Galardini M, Bazzicalupo M, Mengoni A (2011). Plant-bacteria association and symbiosis: Are there common genomic traits in Alphaproteobacteria?. Genes (Basel).

[40] Bulgarelli D, Garrido-Oter R, Münch PC, Weiman A, Dröge J, Pan Y, McHardy AC, Schulze-Lefert P (2015). Structure and function of the bacterial root microbiota in wild and domesticated barley. Cell Host Microbe.

[41] Boukhatem ZF, Merabet C, Tsaki H (2022). Plant growth promoting actinobacteria, the most promising candidates as bioinoculants?. Front. Agron..

[42] Pérez-Jaramillo JE, Carrión VJ, de Hollander M, Raaijmakers JM (2018). The wild side of plant microbiomes. Microbiome.

[43] Zarraonaindia I (2015). The soil microbiome influences grapevine-associated microbiota. MBio.

[44] Thomas P, Sahu PK (2021). Vertical transmission of diverse cultivation-recalcitrant endophytic bacteria elucidated using watermelon seed embryos. Front. Microbiol..

[45] Verma SK, Sahu PK, Kumar K, Pal G, Gond SK, Kharwar RN, White JF (2022). Endophyte roles in nutrient acquisition, root system architecture development and oxidative stress tolerance. Appl. Microbiol..

[46] Kandel SL, Joubert PM, Doty SL (2017). Bacterial endophyte colonization and distribution within plants. Microorganisms.

[47] Morella NM, Weng FCH, Joubert PM, Jessica C, Lindow S, Koskella B (2020). Successive passaging of a plant-associated microbiome reveals robust habitat and host genotype-dependent selection. Proc. Natl. Acad. Sci. USA.

[48] Berg G, Cernava T (2022). The plant microbiota signature of the Anthropocene as a challenge for microbiome research. Microbiome.

[49] Gutierrez A, Grillo MA (2022). Effects of domestication on plant-microbiome interactions. Plant Cell Physiol..

