# Supplementary material for: Metataxonomic analysis of endophytic bacteria of blackberry (Rubus ulmifolius Schott) across tissues and environmental conditions
Source: Sci Rep. 2024 Jun 11;14:13388. doi: 10.1038/s41598-024-64248-5 (PMC11166949; doi:10.1038/s41598-024-64248-5)
Supplement: Supplementary file 1 — Supplementary Information. [file 41598_2024_64248_MOESM1_ESM.docx]

**METATAXONOMIC ANALYSIS OF ENDOPHYTIC BACTERIA OF BLACKBERRY (*Rubus ulmifolius* Schott) ACROSS TISSUES AND ENVIRONMENTAL CONDITIONS**

**Rocío Roca-Couso ^1,2^, José David Flores-Félix ^1,2,3^*,** **Saptarathi Deb^4^, Lucia Giagnoni^4^, Alessandra Tondello^4^, Piergiorgio Stevanato^4^, Andrea Squartini^4^, Paula García-Fraile ^1,2,5^ and Raúl Rivas ^1,2,5^**

**^1^ Department of Microbiology and Genetics, Biology Departmental Building, University of Salamanca, 37007 Salamanca, Spain**

**^2^ Institute for Agribiotechnology Research (CIALE), 37185 Salamanca, Spain**

**^3^ CICS-UBI–Health Sciences Research Centre, University of Beira Interior, 6201-506 Covilhã, Portugal**

**^4^ Department of Agronomy, Animals, Food, Natural Resources, and Environment, DAFNAE University of Padova, 35020 Legnaro (PD), Italy**

**^5^ Associated Unit, University of Salamanca-CSIC (IRNASA), 37008 Salamanca, Spain**

*** Correspondence:** [**jdflores@usal.es**](mailto:jdflores@usal.es) **(J.D.F-F.)**


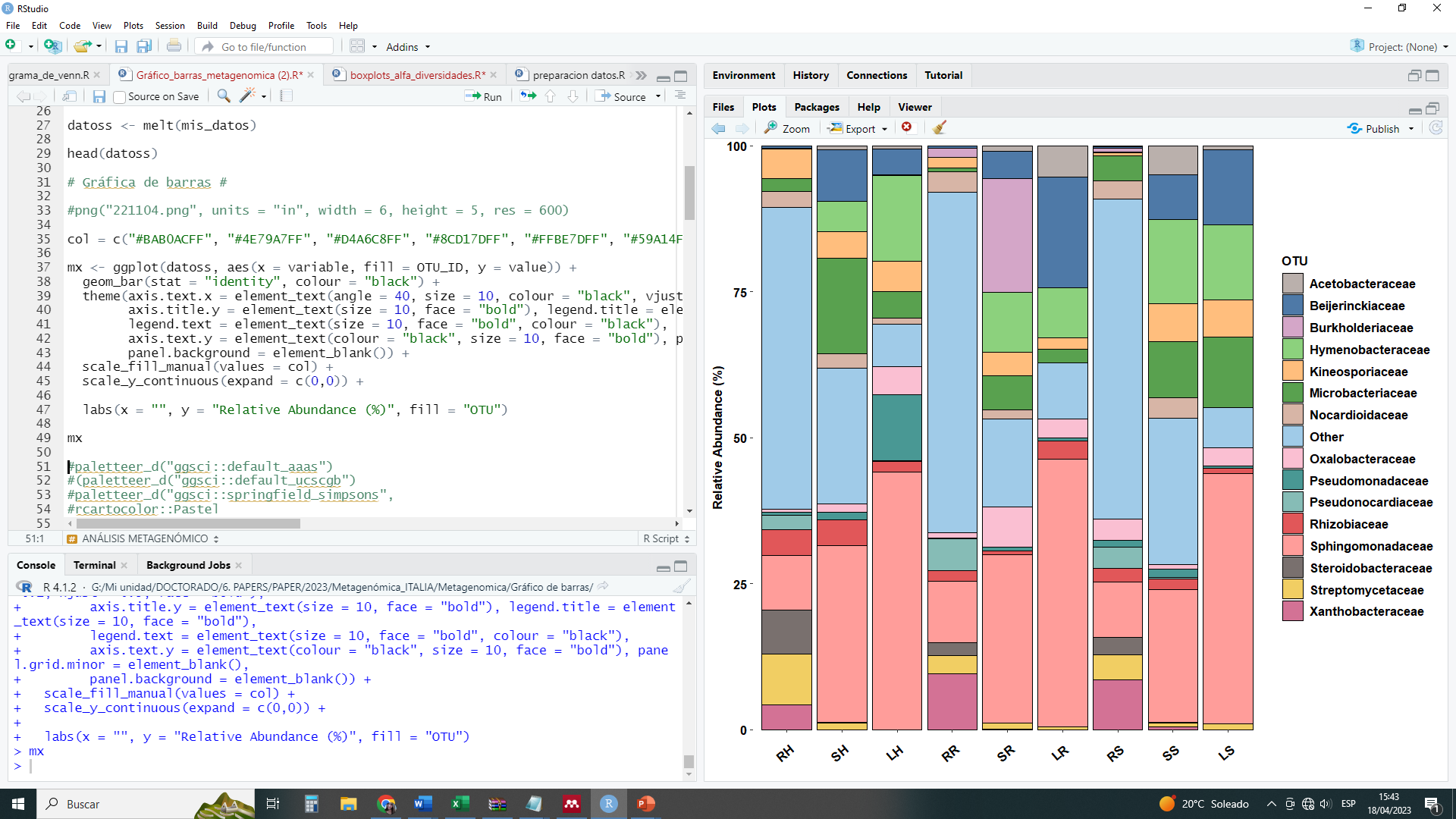


Figure S1. Relative abundance of bacterial family in different anatomic parts of the *Rubus ulmifolius* plant and different conditions. The figure shows bars of roots, stems and leaves in human impacted conditions (RH, SH, LH); of roots, stems and leaves in riverside conditions (RR, SR, LR); and of roots, stems and leaves in standard conditions (RS, SS, LS)

Table S1. Pairwise multiple comparison by Dunn’s test of taxa abundance for samples.

|  | **Pair** | **Z-value** | **P.unadj** | **P.adj** |
| --- | --- | --- | --- | --- |
| **Phylum** | | | | |
| *Acidobacteriota* | LR - RR | -2.426 | 0.015 | 0.046 |
|  | LS - RS | -2.545 | 0.011 | 0.033 |
| *Actinobacteriota* | LR - RR | -2.683 | 0.007 | 0.022 |
| *Bdellovibrionota* | LR - RR | -2.449 | 0.014 | 0.043 |
|  | LH - RH | -2.397 | 0.017 | 0.05 |
| *Chloroflexi* | LS - RS | -2.426 | 0.015 | 0.046 |
|  | LH - RH | -2.397 | 0.017 | 0.05 |
| *Firmicutes* | LH - SH | 2.46 | 0.014 | 0.042 |
| *Gemmatimonadota* | LR - RR | -2.449 | 0.014 | 0.043 |
|  | LS - RS | -2.569 | 0.01 | 0.031 |
| *Myxococcota* | LS - RS | -2.395 | 0.017 | 0.05 |
| *Patescibacteria* | LR - RR | -2.449 | 0.014 | 0.043 |
| *Proteobacteria* | LR - LS | 2.534 | 0.011 | 0.034 |
|  | LR - RR | 2.534 | 0.011 | 0.034 |
| *Planctomycetota* | LR - RR | -2.569 | 0.01 | 0.031 |
|  | LH - RH | -2.562 | 0.01 | 0.031 |
| *Verrucomicrobiota* | LR - RR | -2.449 | 0.014 | 0.043 |
|  | LH - RH | -2.397 | 0.017 | 0.05 |
| **Family** | | | | |
| *Acetobacteraceae* | LR - RR | 2.695 | 0.007 | 0.021 |
| *Beijerinckiaceae* | LR - RR | 2.683 | 0.007 | 0.022 |
|  | LS - RS | 2.534 | 0.011 | 0.034 |
|  | LH - LR | -2.534 | 0.011 | 0.034 |
| *Burkholderiaceae* | LS - RS | -2.426 | 0.015 | 0.046 |
| *Hymenobacteraceae* | LH - RH | 2.562 | 0.01 | 0.031 |
| *Nocardioidaceae* | LR - RR | -2.578 | 0.01 | 0.03 |
|  | LH - LR | 2.449 | 0.014 | 0.043 |
| *Pseudomonadaceae* | LH - LS | 2.395 | 0.017 | 0.05 |
| *Pseudonocardiaceae* | LR - RR | -2.569 | 0.01 | 0.031 |
|  | LS - RS | -2.569 | 0.01 | 0.031 |
| *Steroidobacteraceae* | LH - RH | -2.397 | 0.017 | 0.05 |
|  | LR - RR | -2.449 | 0.014 | 0.043 |
|  | LS - RS | -2.569 | 0.01 | 0.031 |
| *Streptomycetaceae* | LH - RH | -2.562 | 0.01 | 0.031 |
|  | LR - RR | -2.534 | 0.011 | 0.034 |
|  | LH - LS | -2.413 | 0.016 | 0.047 |
| *Xanthobacteraceae* | LR - RR | -2.449 | 0.014 | 0.043 |
|  | LS - RS | -2.729 | 0.006 | 0.019 |

Bidirectional asymptotic significances (2-sided test) are displayed as ±Z-values (Dunn’s pairwise Z test). Pair, indicates the plant sample for roots, stems and leaves in human impacted conditions (RH, SH, LH); of roots, stems and leaves in riverside conditions (RR, SR, LR); and of roots, stems and leaves in standard conditions (RS, SS, LS). p-value, statistical significance level. q-value, Bonferroni correction for multiple tests. Values <0.05 are considered significant.

Table S2. Pairwise multiple comparison by Dunn’s test of alpha diversity indexes values for samples for Figure 2.

| Index | Pair | Z-value | P.unadj | P.adj |
| --- | --- | --- | --- | --- |
| S.obs | LR - RR | -2.683 | 0.007 | 0.022 |
|  | LS - RS | -2.683 | 0.007 | 0.022 |
|  | LH - RH | -2.5 | 0.012 | 0.037 |
| S.chao1 | LR - RR | -2.683 | 0.007 | 0.022 |
|  | LS - RS | -2.683 | 0.007 | 0.022 |
|  | LH - RH | -2.5 | 0.012 | 0.037 |
| S.ACE | LR- RR | -2.683 | 0.007 | 0.022 |
|  | LS - RS | -2.683 | 0.007 | 0.022 |
|  | LH - RH | -2.5 | 0.012 | 0.037 |
| S.Shannon | LR - RR | -2.405 | 0.016 | 0.048 |
|  | LS - RS | -2.47 | 0.014 | 0.041 |
|  | LH - RH | -2.515 | 0.012 | 0.036 |
| S.Simpson | LH - RH | -2.594 | 0.009 | 0.028 |

Bidirectional asymptotic significances (2-sided test) are displayed as ±Z-values (Dunn’s pairwise Z test). Pair, indicates the plant sample for roots, stems and leaves in human impacted conditions (RH, SH, LH); of roots, stems and leaves in riverside conditions (RR, SR, LR); and of roots, stems and leaves in standard conditions (RS, SS, LS). p-value, statistical significance level. q-value, Bonferroni correction for multiple tests. Values <0.05 are considered significant.

Table S3. Predicted metabolic pathways inferred by PICRUSt2 in the bacteria communities of *Rubus ulmifolius*

| Pathway | Root - Standard conditions | | | Stem - Standard conditions | | | Leaves - Standard conditions | | | Root - Riverside conditions | | | Stem - Riverside conditions | | | Leaves - Riverside conditions | | | Root - Human-impacted conditions | | | Stem - Human-impacted conditions | | | Leaves - Human-impacted conditions | | |
| --- | --- | --- | --- | --- | --- | --- | --- | --- | --- | --- | --- | --- | --- | --- | --- | --- | --- | --- | --- | --- | --- | --- | --- | --- | --- | --- | --- |
|  | Hits | Pval | FDR | Hits | Pval | FDR | Hits | Pval | FDR | Hits | Pval | FDR | Hits | Pval | FDR | Hits | Pval | FDR | Hits | Pval | FDR | Hits | Pval | FDR | Hits | Pval | FDR |
| Alanine aspartate and glutamate metabolism | 42 | 0.011 | 0.059 | 35 | 0.014 | 0.066 | 38 | 0.001 | 0.008 | 45 | 0.000 | 0.006 | 41 | 0.003 | 0.018 | 39 | 0.000 | 0.005 | 44 | 0.001 | 0.009 | 41 | 0.001 | 0.008 | 40 | 0.000 | 0.002 |
| Amino sugar and nucleotide sugar metabolism | 97 | 0.005 | 0.033 | 80 | 0.006 | 0.036 | - | - | - | 98 | 0.002 | 0.016 | 89 | 0.012 | 0.061 | - | - | - | 97 | 0.002 | 0.014 | 90 | 0.002 | 0.011 | 76 | 0.036 | 0.140 |
| Aminobenzoate degradation | 26 | 0.033 | 0.149 | - | - | - | - | - | - | 26 | 0.030 | 0.121 | 24 | 0.048 | 0.177 | - | - | - | 26 | 0.023 | 0.104 | - | - | - | - | - | - |
| Arginine and proline metabolism | 60 | 0.002 | 0.021 | 54 | 0.000 | 0.001 | 51 | 0.001 | 0.009 | 58 | 0.008 | 0.040 | 58 | 0.001 | 0.006 | 51 | 0.001 | 0.012 | 57 | 0.010 | 0.052 | 59 | 0.000 | 0.001 | 53 | 0.000 | 0.003 |
| Arginine biosynthesis | 37 | 0.003 | 0.026 | 35 | 0.000 | 0.001 | 30 | 0.010 | 0.057 | 39 | 0.000 | 0.003 | 36 | 0.001 | 0.009 | 29 | 0.024 | 0.104 | 38 | 0.001 | 0.006 | 37 | 0.000 | 0.001 | 31 | 0.006 | 0.036 |
| Benzoate degradation | 73 | 0.000 | 0.000 | 58 | 0.000 | 0.003 | 54 | 0.005 | 0.030 | 73 | 0.000 | 0.000 | 63 | 0.001 | 0.010 | 50 | 0.050 | 0.202 | 70 | 0.000 | 0.001 | 59 | 0.007 | 0.037 | 52 | 0.021 | 0.094 |
| beta-Alanine metabolism | 25 | 0.023 | 0.105 | 21 | 0.028 | 0.107 | 21 | 0.024 | 0.111 | 27 | 0.002 | 0.014 | 23 | 0.038 | 0.147 | - | - | - | 25 | 0.016 | 0.078 | 23 | 0.023 | 0.101 | 21 | 0.028 | 0.119 |
| Biosynthesis of vancomycin group antibiotics | 11 | 0.005 | 0.033 | - | - | - | - | - | - | 11 | 0.005 | 0.028 | 10 | 0.020 | 0.092 | - | - | - | 11 | 0.004 | 0.025 | - | - | - | - | - | - |
| Butanoate metabolism | 78 | 0.000 | 0.002 | 67 | 0.000 | 0.001 | 64 | 0.000 | 0.003 | 79 | 0.000 | 0.001 | 74 | 0.000 | 0.002 | 67 | 0.000 | 0.001 | 78 | 0.000 | 0.001 | 74 | 0.000 | 0.000 | 61 | 0.004 | 0.028 |
| C5-Branched dibasic acid metabolism | - | - | - | - | - | - | - | - | - | 21 | 0.006 | 0.030 | - | - | - | - | - | - | 19 | 0.047 | 0.191 | - | - | - | - | - | - |
| Carbon fixation in photosynthetic organisms | 25 | 0.000 | 0.002 | 24 | 0.000 | 0.000 | 24 | 0.000 | 0.000 | 25 | 0.000 | 0.001 | 25 | 0.000 | 0.000 | 25 | 0.000 | 0.000 | 25 | 0.000 | 0.001 | 25 | 0.000 | 0.000 | 24 | 0.000 | 0.000 |
| Carbon fixation pathways in prokaryotes | - | - | - | - | - | - | - | - | - | 69 | 0.034 | 0.123 | 65 | 0.029 | 0.122 | 59 | 0.011 | 0.059 | 75 | 0.000 | 0.005 | 65 | 0.011 | 0.053 | 59 | 0.012 | 0.062 |
| Carotenoid biosynthesis | - | - | - | - | - | - | - | - | - | - | - | - | - | - | - | 14 | 0.046 | 0.191 | - | - | - | - | - | - | - | - | - |
| Citrate cycle (TCA cycle) | 43 | 0.004 | 0.031 | 41 | 0.000 | 0.001 | 39 | 0.000 | 0.003 | 45 | 0.000 | 0.006 | 43 | 0.000 | 0.004 | 42 | 0.000 | 0.000 | 45 | 0.000 | 0.005 | 43 | 0.000 | 0.002 | 41 | 0.000 | 0.001 |
| Cyanoamino acid metabolism | - | - | - | - | - | - | 12 | 0.029 | 0.128 | - | - | - | - | - | - | - | - | - | - | - | - | - | - | - | - | - | - |
| Cysteine and methionine metabolism | - | - | - | - | - | - | - | - | - | - | - | - | 65 | 0.029 | 0.122 | - | - | - | - | - | - | - | - | - | 60 | 0.007 | 0.041 |
| Drug metabolism - cytochrome P450 | 7 | 0.036 | 0.152 | - | - | - | - | - | - | 7 | 0.035 | 0.123 | - | - | - | - | - | - | - | - | - | - | - | - | - | - | - |
| Drug metabolism - other enzymes | - | - | - | 15 | 0.017 | 0.072 | 15 | 0.015 | 0.074 | 17 | 0.023 | 0.098 | - | - | - | 15 | 0.016 | 0.071 | - | - | - | 16 | 0.019 | 0.087 | 15 | 0.017 | 0.076 |
| Fatty acid biosynthesis | 24 | 0.006 | 0.036 | 23 | 0.000 | 0.003 | 23 | 0.000 | 0.003 | 24 | 0.005 | 0.029 | 24 | 0.001 | 0.010 | 22 | 0.001 | 0.012 | 24 | 0.004 | 0.025 | 24 | 0.001 | 0.005 | 22 | 0.001 | 0.011 |
| Fatty acid degradation | 22 | 0.004 | 0.031 | 20 | 0.001 | 0.011 | 20 | 0.001 | 0.009 | 22 | 0.004 | 0.023 | 22 | 0.001 | 0.009 | 20 | 0.001 | 0.012 | 22 | 0.003 | 0.019 | 20 | 0.009 | 0.045 | 21 | 0.000 | 0.003 |
| Fluorobenzoate degradation | - | - | - | 10 | 0.016 | 0.072 | 10 | 0.015 | 0.074 | - | - | - | - | - | - | 10 | 0.016 | 0.071 | - | - | - | 10 | 0.043 | 0.156 | - | - | - |
| Fructose and mannose metabolism | 70 | 0.007 | 0.043 | 56 | 0.025 | 0.099 | 54 | 0.049 | 0.197 | 70 | 0.006 | 0.031 | 67 | 0.003 | 0.018 | - | - | - | 69 | 0.007 | 0.038 | 68 | 0.000 | 0.003 | 57 | 0.015 | 0.073 |
| Furfural degradation | - | - | - | 6 | 0.014 | 0.066 | 66 | 0.000 | 0.001 | - | - | - | 6 | 0.035 | 0.137 | 6 | 0.013 | 0.070 | - | - | - | - | - | - | - | - | - |
| Galactose metabolism | 43 | 0.037 | 0.152 | - | - | - | - | - | - | 47 | 0.001 | 0.011 | 41 | 0.023 | 0.105 | - | - | - | 42 | 0.044 | 0.183 | 44 | 0.001 | 0.006 | - | - | - |
| Glycine serine and threonine metabolism | 73 | 0.004 | 0.030 | 67 | 0.000 | 0.001 | - | - | - | 73 | 0.003 | 0.020 | 73 | 0.000 | 0.002 | 68 | 0.000 | 0.000 | 75 | 0.000 | 0.005 | 71 | 0.000 | 0.002 | 67 | 0.000 | 0.001 |
| Glycolysis / Gluconeogenesis | 69 | 0.000 | 0.004 | 58 | 0.000 | 0.003 | 57 | 0.001 | 0.006 | 70 | 0.000 | 0.001 | 66 | 0.000 | 0.002 | 59 | 0.000 | 0.002 | 69 | 0.000 | 0.002 | 67 | 0.000 | 0.000 | 59 | 0.000 | 0.002 |
| Glyoxylate and dicarboxylate metabolism | 73 | 0.000 | 0.000 | 69 | 0.000 | 0.000 | 67 | 0.000 | 0.000 | 73 | 0.000 | 0.000 | 72 | 0.000 | 0.000 | 70 | 0.000 | 0.000 | 74 | 0.000 | 0.000 | 71 | 0.000 | 0.000 | 68 | 0.000 | 0.000 |
| Histidine metabolism | 28 | 0.019 | 0.092 | 25 | 0.006 | 0.036 | 26 | 0.002 | 0.012 | 28 | 0.017 | 0.076 | 27 | 0.011 | 0.057 | 25 | 0.005 | 0.036 | 30 | 0.001 | 0.011 | 29 | 0.001 | 0.004 | 25 | 0.006 | 0.038 |
| Lysine biosynthesis | 34 | 0.003 | 0.026 | 27 | 0.022 | 0.090 | 39 | 0.000 | 0.003 | - | - | - | - | - | - | - | - | - | - | - | - | 31 | 0.027 | 0.106 | - | - | - |
| Lysine biosynthesis | - | - | - | - | - | - | - | - | - | 35 | 0.001 | 0.009 | 33 | 0.001 | 0.010 | - | - | - | 34 | 0.002 | 0.014 | 32 | 0.002 | 0.011 | - | - | - |
| Nitrogen metabolism | 40 | 0.045 | 0.176 | 35 | 0.014 | 0.066 | - | - | - | 41 | 0.020 | 0.086 | - | - | - | 35 | 0.012 | 0.066 | 40 | 0.029 | 0.127 | - | - | - | 36 | 0.007 | 0.039 |
| Oxidative phosphorylation | 93 | 0.000 | 0.000 | 83 | 0.000 | 0.000 | 78 | 0.000 | 0.000 | 93 | 0.000 | 0.000 | 89 | 0.000 | 0.000 | 84 | 0.000 | 0.000 | 93 | 0.000 | 0.000 | 81 | 0.000 | 0.001 | 87 | 0.000 | 0.000 |
| Pantothenate and CoA biosynthesis | - | - | - | - | - | - | 46 | 0.001 | 0.008 | 30 | 0.010 | 0.046 | - | - | - | - | - | - | - | - | - | - | - | - | - | - | - |
| Pentose phosphate pathway | 51 | 0.017 | 0.086 | 45 | 0.003 | 0.021 | - | - | - | 53 | 0.003 | 0.020 | 50 | 0.004 | 0.021 | 44 | 0.006 | 0.036 | 53 | 0.002 | 0.014 | 49 | 0.003 | 0.016 | 45 | 0.003 | 0.023 |
| Phenylalanine metabolism | 51 | 0.000 | 0.001 | 36 | 0.043 | 0.157 | 39 | 0.003 | 0.018 | 51 | 0.000 | 0.001 | - | - | - | - | - | - | 43 | 0.036 | 0.153 | 40 | 0.032 | 0.119 | 36 | 0.043 | 0.161 |
| Phenylalanine tyrosine and tryptophan biosynthesis | 47 | 0.001 | 0.017 | 41 | 0.001 | 0.005 | - | - | - | 50 | 0.000 | 0.001 | 44 | 0.002 | 0.015 | 37 | 0.015 | 0.071 | 46 | 0.002 | 0.014 | 44 | 0.001 | 0.006 | 42 | 0.000 | 0.003 |
| Photosynthesis | 54 | 0.000 | 0.000 | 41 | 0.000 | 0.001 | 52 | 0.000 | 0.000 | 41 | 0.032 | 0.123 | 54 | 0.000 | 0.000 | 53 | 0.000 | 0.000 | 41 | 0.023 | 0.104 | 52 | 0.000 | 0.000 | 41 | 0.000 | 0.002 |
| Porphyrin metabolism | 67 | 0.002 | 0.021 | 62 | 0.000 | 0.001 | 60 | 0.000 | 0.001 | 67 | 0.002 | 0.014 | 66 | 0.000 | 0.003 | 62 | 0.000 | 0.000 | 67 | 0.001 | 0.009 | 65 | 0.000 | 0.001 | 64 | 0.000 | 0.000 |
| Propanoate metabolism | 61 | 0.013 | 0.068 | 51 | 0.011 | 0.058 | 49 | 0.025 | 0.114 | 64 | 0.001 | 0.011 | 60 | 0.002 | 0.012 | 52 | 0.005 | 0.035 | 63 | 0.001 | 0.012 | 61 | 0.000 | 0.002 | - | - | - |
| Purine metabolism | - | - | - | 83 | 0.041 | 0.153 | 82 | 0.042 | 0.176 | - | - | - | - | - | - | 85 | 0.016 | 0.071 | - | - | - | 93 | 0.026 | 0.103 | 88 | 0.005 | 0.033 |
| Pyrimidine metabolism | - | - | - | - | - | - | 75 | 0.000 | 0.001 | - | - | - | - | - | - | 52 | 0.018 | 0.081 | - | - | - | - | - | - | 51 | 0.035 | 0.140 |
| Pyruvate metabolism | 86 | 0.001 | 0.014 | 75 | 0.000 | 0.001 | - | - | - | 86 | 0.001 | 0.008 | 82 | 0.000 | 0.004 | 78 | 0.000 | 0.000 | 86 | 0.000 | 0.005 | 83 | 0.000 | 0.001 | 78 | 0.000 | 0.000 |
| Selenocompound metabolism | 16 | 0.013 | 0.068 | 15 | 0.003 | 0.021 | 15 | 0.003 | 0.017 | 16 | 0.012 | 0.055 | 16 | 0.004 | 0.023 | 15 | 0.003 | 0.020 | 16 | 0.010 | 0.052 | 15 | 0.012 | 0.059 | 15 | 0.003 | 0.023 |
| Starch and sucrose metabolism | 58 | 0.001 | 0.014 | 52 | 0.000 | 0.001 | 49 | 0.001 | 0.008 | 57 | 0.002 | 0.015 | 56 | 0.000 | 0.004 | 48 | 0.002 | 0.019 | 55 | 0.006 | 0.036 | 53 | 0.001 | 0.009 | 49 | 0.001 | 0.011 |
| Steroid degradation | - | - | - | 9 | 0.009 | 0.051 | 9 | 0.008 | 0.048 | - | - | - | 9 | 0.032 | 0.128 | 9 | 0.009 | 0.050 | - | - | - | 9 | 0.024 | 0.101 | 9 | 0.009 | 0.049 |
| Taurine and hypotaurine metabolism | - | - | - | - | - | - | - | - | - | - | - | - | - | - | - | - | - | - | - | - | - | 21 | 0.000 | 0.002 | 15 | 0.032 | 0.135 |
| Terpenoid backbone biosynthesis | 21 | 0.002 | 0.020 | 20 | 0.000 | 0.002 | 20 | 0.000 | 0.002 | 21 | 0.002 | 0.014 | 21 | 0.000 | 0.004 | 19 | 0.001 | 0.009 | 21 | 0.001 | 0.011 | 28 | 0.025 | 0.102 | 20 | 0.000 | 0.002 |
| Toluene degradation | 28 | 0.035 | 0.152 | - | - | - | - | - | - | 28 | 0.032 | 0.123 | - | - | - | - | - | - | - | - | - | - | - | - | - | - | - |
| Tryptophan metabolism | 30 | 0.038 | 0.152 | 27 | 0.009 | 0.049 | 26 | 0.016 | 0.080 | 30 | 0.034 | 0.123 | 29 | 0.020 | 0.092 | 27 | 0.008 | 0.048 | - | - | - | 39 | 0.028 | 0.106 | 27 | 0.009 | 0.047 |
| Tyrosine metabolism | - | - | - | 36 | 0.022 | 0.090 | 35 | 0.033 | 0.142 | 42 | 0.041 | 0.142 | 40 | 0.029 | 0.122 | - | - | - | - | - | - | 33 | 0.015 | 0.072 | 37 | 0.011 | 0.058 |
| Ubiquinone and other terpenoid-quinone biosynthesis | - | - | - | 32 | 0.004 | 0.025 | 31 | 0.007 | 0.043 | - | - | - | 35 | 0.006 | 0.031 | 31 | 0.008 | 0.048 | - | - | - | 17 | 0.000 | 0.001 | 29 | 0.039 | 0.151 |
| Valine leucine and isoleucine biosynthesis | 17 | 0.000 | 0.005 | 17 | 0.000 | 0.000 | 16 | 0.000 | 0.001 | 17 | 0.000 | 0.004 | 17 | 0.000 | 0.002 | 15 | 0.001 | 0.009 | 17 | 0.000 | 0.004 | 44 | 0.000 | 0.001 | 15 | 0.001 | 0.008 |
| Valine leucine and isoleucine degradation | 47 | 0.000 | 0.002 | 42 | 0.000 | 0.001 | 41 | 0.000 | 0.001 | 48 | 0.000 | 0.001 | 44 | 0.000 | 0.004 | 41 | 0.000 | 0.001 | 48 | 0.000 | 0.001 | 12 | 0.001 | 0.006 | 39 | 0.001 | 0.008 |
| Vitamin B6 metabolism | 12 | 0.003 | 0.026 | 10 | 0.016 | 0.072 | 10 | 0.015 | 0.074 | 12 | 0.003 | 0.020 | 12 | 0.001 | 0.010 | 11 | 0.002 | 0.019 | 11 | 0.022 | 0.104 | - | - | - | 10 | 0.016 | 0.076 |
| Xylene degradation | - | - | - | 23 | 0.013 | 0.066 | - | - | - | - | - | - | 24 | 0.048 | 0.177 | - | - | - | - | - | - | - | - | - | - | - | - |
